# Characterization and Distribution of Agar-degrading *Steroidobacter agaridevorans* sp. nov., Isolated from Rhizosphere Soils

**DOI:** 10.1264/jsme2.ME20136

**Published:** 2021-03-13

**Authors:** Makoto Ikenaga, Machi Kataoka, Xuan Yin, Aya Murouchi, Masao Sakai

**Affiliations:** 1 Research Field in Agriculture, Agriculture Fisheries and Veterinary Medicine Area, Kagoshima University, 1–21–24, Korimoto, Kagoshima, 890–0065, Japan; 2 The United Graduate School of Agricultural Sciences, Kagoshima University, 1–21–24, Korimoto, Kagoshima, 890–0065, Japan; 3 Faculty of Agriculture, Kagoshima University, 1–21–24, Korimoto, Kagoshima, 890–0065, Japan; 4 Graduate School of Agriculture, Kagoshima University, 1–21–24, Korimoto, Kagoshima, 890–0065, Japan

**Keywords:** agar-degrading, diffusible metabolite, ecological distribution, novel species, *Steroidobacter agaridevorans*

## Abstract

The environment of plant rhizosphere soil differs from that of non-rhizosphere soil due to the secretion of mucilage polysaccharides from the roots. This environment is regarded as one of the preferential habitats for agar-degrading bacteria. In a previous study, agar-degrading *Steroidobacter agariperforans* KA5-B^T^ was isolated from agar-enriched agricultural soil using diffusible metabolites from *Rhizobiales* bacteria. Based on the hypothesis that similar characteristic bacteria still exist in the rhizosphere, isolation was performed using rhizosphere soils. Agar-degrading SA29-B^T^ and YU21-B were isolated from onion and soybean rhizosphere soils. The 16S rRNA genes of these strains showed ≥98.7% identities with the most closely related strain KA5-B^T^. However, differences were noted in polysaccharide utilization, and average nucleotide identities were <95–96% against strain KA5-B^T^, indicating that they are different species from *S. agariperforans* KA5-B^T^. To investigate the distribution of bacterial sequences affiliated with novel strains, a primer set was designed and a meta-analysis of the 16S rRNA gene was performed. Sequences were widely distributed in rhizospheres throughout Japan, but varied in plant- and region-dependent manners. Regarding phenotypic characterization, distinguishable features were observed in growth temperatures, pH, and dominant fatty acids. SA29-B^T^ and YU21-B grew at 15–40°C and pH 6.0–12 and contained C_16:0_ as the dominant cell fatty acid, whereas KA5-B^T^ showed no growth at 40°C and pH 12 and contained a moderate amount of C_16:0_. Based on these characteristics, SA29-B^T^ (JCM 333368^T^=KCTC 72223^T^) and YU21-B (JCM 333367=KCTC 72222) represent novel species in the genus *Steroidobacter*, for which the name *Steroidobacter agaridevorans* sp. nov. is proposed.

*Steroidobacter* is a bacterial genus within the family *Steroidobacteraceae* in the order *Nevskiales* of *Gammaproteobacteria*. *Steroidobacter denitrificans* FS^T^ was initially proposed as the type species in this novel genus (Fahrbach *et al.*, 2008), and two novel species, *Steroidobacter agariperforans* KA5-B^T^ (Sakai *et al.*, 2014) and *Steroidobacter soli* JW-3^T^ (Huang *et al.*, 2019) were subsequently approved. “*Steroidobacter flavus*” CPCC 100154 (Gong *et al.*, 2016) and “*Steroidobacter cummioxidans*” 35Y (Sharma *et al.*, 2018) have since been proposed. *Povalibacter uvarum* Zumi 37^T^ (Nogi *et al.*, 2014) was also approved as a closely related novel genus in the same family. We previously isolated *S. agariperforans* KA5-B^T^ from agricultural soil collected in Fukuoka prefecture, Japan after an integrated incubation by mixing dissolved agar (Sakai *et al.*, 2014). Careful investigations revealed that this strain required diffusible metabolites from companion bacteria belonging to the order *Rhizobiales* for its regular growth.

The environment of plant rhizosphere soil differs from that of non-rhizosphere soil due to the secretion of mucilage polysaccharides from the roots. In addition, the relative abundance of *Rhizobiales* bacteria is higher in rhizosphere soils and roots than in non-rhizosphere soils (Garrido-Oter *et al.*, 2018). This environment appears to be the optimal habitat for agar-degrading bacteria, such as strain KA5-B^T^. Agar-degrading bacteria have been isolated from rhizosphere soils, including *Paenibacillus* spp. from the spinach rhizosphere (Hosoda *et al.*, 2003) and *Asticcacaulis* sp. SA7 from the plant rhizosphere (Hosoda and Sakai, 2006). However, these studies only used agar plate media for isolation, and, thus, bacteria requiring co-cultivations were not included. Therefore, KA5-B^T^ like agar-degrading bacteria are still present and may be isolated from rhizosphere soils.

Based on the hypothesis that bacteria with similar characteristics may be obtained from the rhizosphere, isolation was performed on rhizosphere soils using an agar medium containing diffusible metabolites obtained from *Rhizobiales* bacteria. Two agar-degrading bacteria, strains SA29-B^T^ and YU21-B, were successfully isolated from onion (*Allium cepa*) and soybean (*Glycine max*) rhizosphere soils.

The aim of this present study was to characterize the taxonomic status of strains SA29-B^T^ and YU21-B in the genus *Steroidobacter* using polyphasic approaches, and identified as a novel species. We also investigated the bacterial sequences affiliated with these novel strains in plant rhizospheres throughout Japan, including non-rhizosphere soils, to obtain basic information on their distribution.

## Materials and Methods

### Soil collection and integrated incubation of agar-degrading bacteria

Soybean (*Glycine max*) and onion (*Allium cepa*) plants were collected from agricultural fields on May 15^th^, 2003 in Sanuki city, Kagawa prefecture (34°15′28″N, 134°10′54″E) and on June 21^th^, 2003 in Ashikita county, Kumamoto prefecture (32°15′49″N, 130°29′39″E), respectively. Each rhizosphere soil sample was collected from soybean and onion roots by aerial shaking. Dissolved agar was mixed into approximately 100‍ ‍g of the respective soils to a final concentration of 1.0% (w/w). Water content was adjusted to 50% ([w/w], water/dry soil). An integrated incubation was performed at 30°C for 14‍ ‍d to enrich agar-degrading bacteria. Some soil was suspended in 10‍ ‍mM phosphate buffer solution (pH 7.0) and serially diluted. Aliquots of the diluted suspension were spread on PSG agar plates (1.7‍ ‍g L^–1^ peptone, 0.3‍ ‍g L^–1^ soytone, 0.25‍ ‍g L^–1^ glucose, 0.25‍ ‍g L^–1^ K_2_HPO_4_, and 0.5‍ ‍g L^–1^ NaCl) containing solidified 1.5% agar and then incubated at 30°C for 10 d.

### Isolation of agar-degrading bacteria using diffusible metabolites prepared from *Rhizobiales* companions

After the incubation, a colony, appearing in a depressed position on agar medium due the degradation of agar, was obtained from each of the soybean and onion rhizosphere soil samples. Each colony was picked up and streaked on a PSG agar plate for isolation. However, similar to KA5-B^T^ (Sakai *et al.*, 2014), two different colonies were observed in both samples. One colony was isolated by repeating streaking, whereas the other was not or a only few small colonies were observed at the site of inoculation with a large number of cells. The small colonies were not observed with subsequent streaking. In the present study, the former and latter isolates obtained from soybean and onion rhizosphere soils were named strains SA29-A and SA29-B^T^ and strains YU21-A and YU21-B respectively. Neither SA29-A nor YU21-A exhibited agar-degrading abilities.

To obtain sufficient numbers of SA29-B^T^ and YU21-B cells, the culture supernatants of SA29-A and YU21-A, which were cultured in PSG liquid medium, were added to PSG liquid medium at a final volume of 10% (v/v). The mixture (PSGS) was sterilized at 121°C for 20‍ ‍min and solidified with 1.5% agar. The small colonies of strains SA29-B^T^ and YU21-B were streaked on PSGS agar plates prepared using the supernatants of strains SA29-A and YU21-A, respectively. After an incubation at 30°C, sufficient amounts of agar-degrading colonies were successfully obtained on PSGS plates.

### Sequencing of bacterial 16S rRNA genes and a phylogenetic analysis

Almost the full-length bacterial 16S rRNA genes of four strains, SA29-A, SA29-B^T^, YU21-A, and YU21-B, were amplified with the 27f and 1492r primer sets (Ikenaga *et al.*, 2002). After amplification, PCR products were purified using a QIAquick PCR purification kit (Qiagen), and the 16S rRNA gene was partially sequenced with internal primers with an ABI 3500xl genetic analyzer (Life Technologies Japan). BigDye terminator v3.1 was used for cycle sequencing (Life Technologies Japan). The resultant 16S rRNA gene sequences of the four strains were used in a similarity search against the sequences in the DNA database using the BLAST program (https://blast.ncbi.nlm.nih.gov/Blast.cgi).

Multiple alignments were performed with the closest relatives using CLUSTAL W built in MEGA software version 7.0.20 (Tamura *et al.*, 2011) to investigate the phylogenetic positions of strains SA29-B^T^ and YU21-B. Phylogenetic trees were constructed based on 1,000 resamplings using three different algorithms; neighbor-joining (Saitou and Nei, 1987), maximum-parsimony (Fitch, 1971), and maximum-likelihood (Felsenstein, 1981).

### Polysaccharide-degrading ability

The polysaccharide-degrading abilities of strains SA29-B^T^ and YU21-B were investigated using the following polysaccharides and a comparison with the most closely related strain, KA5-B^T^; arabinan, wheat arabinoxylan, galactomannan, xyloglucan, debranched arabinan, pectic galactan, larch arabinogalactan, and polygalacturonic acid (1.0‍ ‍g L^–1^ each), agar, dextrin, and starch (2.0‍ ‍g L^–1^ each), carboxymethyl cellulose (CM-cellulose), and chitin and xylan (5.0‍ ‍g L^–1^ each) were individually dissolved in HMM medium (0.125‍ ‍g L^–1^ Na_2_HPO_4_·2H_2_O, 0.25‍ ‍g L^–1^ Na_2_SO, 0.32‍ ‍g L^–1^ NH_4_Cl, 0.18‍ ‍g L^–1^ MgSO_4_, 0.0067‍ ‍g L^–1^ FeCl_3_·6H_2_O, and 0.013‍ ‍g‍ ‍L^–1^ CaCl_2_·2H_2_O) solidified with 1.5% gellan gum. The cells of each strain were prepared by culturing in PSGS liquid medium.

Regarding the experimental procedure, the same method as that to evaluate the polysaccharide-degrading ability of strain KA5-B^T^ was used (Sakai *et al.*, 2014). In brief, rectangular-shaped filter paper was soaked in sterilized supernatants including the diffusible metabolites of each companion cultured in PSG liquid medium. The suspended cells of SA29-B^T^ and YU21-B were inoculated on gellan gum plates by streaking in parallel along the long side of the filter paper and then incubated at 30°C for 14 d.

The degradation of agar, starch, and dextrin was detected using Lugol’s staining technique (Ruijssenaars and Hartmans, 2001), while that of other polysaccharides was visualized by Congo red staining (Suyama *et al.*, 1993) followed by decolorization with 1.0‍ ‍M NaCl solution. Degradation abilities by both staining methods were confirmed based on the appearance of a clear zone surrounding the bacterial colony.

### Whole-genome sequencing and average nucleotide identity analysis

The whole-genome sequencing of strains SA29-B^T^ and YU21-B was performed by the Global Catalogue of Microorganisms (GCM) 10K type strain sequencing project (Wu and Ma, 2019). DNA extraction was conducted using the CTAB DNA extraction protocol (available at http://gcm.wdcm.org/typestrain/material/sop1.pdf), and high-throughput sequencing with the Illumina platform.

An average nucleotide identity (ANI) analysis was performed by TechnoSuruga Laboratory (https://www.tecsrg.co.jp) using the whole-genome sequences of three strains registered to GenBank with GCA_009932555.1, GCA_009932515.1, and GCA_004138335.1 for strains SA29-B^T^, YU21-B, and KA5-B^T^, respectively. An online ANI calculator (http://enve-omics.ce.gatech.edu/ani/index) was used (Rodrigues-R, L.M., and Konstantinidis, K.T. 2016. The enveomics collection: a toolbox for specialized analyses of microbial genomes and metagenomes. *PeerJ Preprints* **4**: e1900v1), and the analysis was conducted on all combinations.

### Design of the primer set to amplify bacterial sequences affiliated with novel strains

To investigate the ecological distribution of the bacterial sequences affiliated with strains SA29-B^T^ and YU21-B in plant rhizosphere soils, a primer set was designed by performing multiple alignments of the 16S rRNA gene sequences of the two strains and the closest relatives using CLUSTAL W. *S. agariperforans* KA5-B^T^ was also considered to be the same targeting group with two novel strains due to its close phylogenetic position and similar feature of requiring co-cultivation. Based on alignment data, primer-designable sequences were identified that distinguished the two novel strains and *S. agariperforans* KA5-B^T^ from the other species in the genera *Steroidobacter* and *Povalibacter* and other species in the order *Nevskiales*. Therefore, a primer set common to strains SA29-B^T^, YU21-B, and *S. agariperforans* KA5-B^T^ was designed using Primer-Blast (https://www.ncbi.nlm.nih.gov/tools/primer-blast/index.cgi?LINK_LOC=BlastHome). It is important to note that distinguishable DNA bases among the three strains were ensured in the sequences between the forward and reverse primers. Therefore, the forward and reverse primers designed were named KUSBf (5′-CTGTTAGTGGGGGACAACCAA-3′, corresponding to positions 135–155 on the complete 16S rRNA gene sequence of *Escherichia coli*) and KUSBr (5′-GCGACGTGTTAGGTCGCA-3′, corresponding to positions 469–452 on the complete sequence of *E. coli*), respectively. Alignment data for designing the primers are shown in supplementary Fig. S1.

### Ecological distribution

Plant rhizosphere soils were collected from agricultural fields in 2020. The rhizosphere soils of garlic (*Allium sativum*), onion (*Allium cepa*) and potato (*Solanum tuberosum*), soybean (*Glycine max*) and tomato (*Solanum lycopersicum*), wax gourd (*Benincasa hispidia* var. *Chieh-qua*), sugarcane (*Saccharum officinarum*), and sweet potato (*Ipomoea batatas*) were collected on May 31^th^ in Towada city, Aomori prefecture (40°61′27″N, 141°20′59″E), on May 23th in Tsuchiura city, Ibaragi prefecture (36°06′67″N, 140°20′00″E), on May 17^th^ in Unzen city, Nagasaki prefecture (32°83′33″N, 130°18′33″E), on Jul 13^th^ in Kagoshima city, Kagoshima prefecture (31°60′00″N, 130°55′00″E), and on Jun 20^th^ in Nishiharacho Nakagami county, Okinawa prefecture (26°39′60″N, 127°74′44″E), respectively. Non-rhizosphere soils were also collected from the same fields in Ibaragi, Nagasaki, and Kagoshima prefectures. The soil types collected from Aomori, Ibaragi, and Kagoshima were brown lowland soil, while those from Nagasaki and Okinawa were andosol and Red and Yellow soil, respectively.

Soil samples were used for DNA extraction in duplicate using the FastDNA SPIN Kit for Soil (MP Biomedicals) with a phenol/chloroform treatment. 16S rRNA genes were initially amplified with the bacterial primer sets of 27f (Ikenaga *et al.*, 2002) and 517r (Ikenaga *et al.*, 2003). PCR conditions were as follows: at 94°C for 3‍ ‍min, followed by 30 cycles at 94°C for 1‍ ‍min, 55°C for 1‍ ‍min, and 72°C for 1.5‍ ‍min, with a final extension step at 72°C for 8‍ ‍min. Aliquots of the products were used for nested PCR with the same amplification conditions using the KUSBf and KUSBr primer sets.

The NGS analysis for amplified regions was performed using a paired-end method with MiSeq (Illumina). Adapter sequences followed by index sequences were amplifically attached for the respective products. The Qiime pipeline (Caporaso *et al.*, 2010) was used in the analysis to remove low quality and chimeric sequences. On the other hand, output data were not compiled into representative OTU sequences because the recognition of single nucleotide differences was important. The NGS analysis was performed by Bioengineering Lab. (http://www.gikenbio.com/).

### Macroscopic and microscopic observations

Macroscopic and microscopic observations were performed to characterize the strains SA29-B^T^ and YU21-B. Both stains were cultured on PSGS gellan gum plates at 30°C for 5 d. This culture period approximately corresponded to the beginning of the stationary growth phase based on colony formation. Motility was examined by phase-contrast microscopy (Olympus). Gram staining was conducted after staining using the Hucker method (Murray *et al.*, 1994). Cell morphology, spore formation, and cell size were examined using the field emission scanning electron microscope (FE-SEM) S-4100H (Hitachi High Technologies). The strains cultured at 30°C for 14‍ ‍d, which approximately corresponded to the death phase, were also used to confirm spore formation. In FE-SEM observations, the cells of both strains were washed with 10‍ ‍mM phosphate buffer (pH 7.0) and placed on glass micro covers. After sequential dehydration and freeze-drying, cells were metal-coated with platinum and observed under the high vacuum mode at 10 kV.

### Physiological analysis

In analyses of physiological characteristics, anaerobic growth, catalase and cytochrome oxidase activities, and growth ranges at different temperatures, pH, and NaCl concentrations were investigated. Strains were inoculated on PSGS gellan gum plates and incubated at 30°C under anaerobic conditions after being packed in an AnaeroPouch bag (Mitsubishi Gas Chemical). Anaerobic growth was assessed based on colony formation. Catalase and cytochrome oxidase activities were examined using the cells grown on gellan gum plates and incubated at 30°C for 5 d. Catalase and cytochrome oxidase activities were confirmed by the formation of bubbles in a 3% H_2_O_2_ solution and using oxidase-testing paper (Nissui Pharmaceutical), respectively. Growth ranges at different temperatures were also examined on PSGS gellan gum plates. Temperature ranges were at intervals of 2°C from 10–42°C, and at intervals of 1°C around the optimum growth temperature. The optimal temperature was identified by a comparison of the colony-forming rate. The ranges of pH and NaCl concentrations were examined in PSGS liquid medium. pH and NaCl concentrations ranged from pH 5.0–13.0 at intervals of 0.5, and 0–4% (w/v) at intervals of 1%, respectively. Turbidity by OD_660_ was used for measurements. Optimal pH and NaCl concentrations were assessed based on the proliferation rate.

### Chemotaxonomic analysis

Cells cultured in PSGS liquid medium until the beginning of the stationary growth phase were used for the whole-cell fatty acid profile analyses of major quinone. Whole-cell fatty acids and quinones were extracted with chloroform/methanol (2:1‍ ‍[v/v]) and purified using the method described by Hiraishi *et al.* (1996). After purification, whole-cell fatty acids and quinones were analyzed using a GC-based Microbial Identification System (MIDI) and Shimadzu LC-10 HPLC system (Shimadzu), respectively. These procedures were the same as those used to examine the most closely related strain KA5-B^T^.

The G+C content (mol%) of genomic DNA was obtained from the genomic DNA GCM type strain genome database (http://gcm.wdcm.org/typestrain/species.jsp); SA29-B^T^, YU21-B, and KA5-B^T^ were from GCM10011488, GCM10011487, and GCM10006637, respectively.

### Substrate utilization

To investigate substrate utilization, cells cultured under optimum conditions were suspended in 10‍ ‍mM sodium phosphate buffer (pH=7.0). Portions of the cell suspension were dispensed into the respective holes of a Biolog GN2 microplate (Biolog) and the microplate was incubated at 30°C. After the incubation, utilization levels were classified into three categories: positive (+), weakly positive (±), and negative (–) based on visual observations. This method was also used to assess substrate utilization by KA5-B^T^.

### Nucleotide sequence accession number

The GenBank/EMBL/DDBJ accession numbers for the 16S rRNA gene sequences of strains SA29-B^T^, YU21-B, SA29-A, and YU21-A are AB174845, AB174846, LC489411, and LC489410, respectively. The assembly numbers of the whole-genome sequences of strains SA29-B^T^ and YU21-B^T^ are ASM993255v1 and ASM993251v1, respectively. Nucleotide sequences obtained in meta 16S amplicon sequencing prepared with KUSBf and KUSBr were registered to Genebank under the accession number PRJDB10611.

## Results and Discussion

### Phylogenetic positions of strains SA29-A, SA29-B^T^, YU21-A, and YU21-B

The 16S rRNA gene sequences of strains SA29-A, YU21-A, SA29-B^T^, and YU21-B were almost completely elucidated to investigate phylogenetic positions. Strains SA29-A and YU21-A grew on PSG agar medium, but did not exhibit any agar-degrading ability. On the other hand, SA29-B^T^ and YU21-B, obtained as companions of SA29-A and YU21-A, required the diffusible metabolites of strains SA29-A and YU21-A for regular growth and exhibited agar-degrading abilities. This result was similar to the relationship reported between strains KA5-A and KA5-B^T^ (Sakai *et al.*, 2014), indicating that rhizosphere soils are one of the preferential habitats for agar-degrading bacteria, such as KA5-B^T^.

**Fig. 1.**
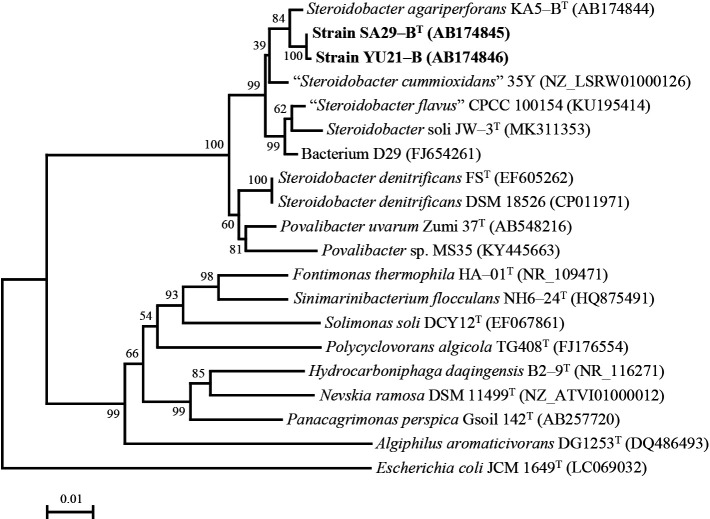
Neighbor-joining tree (Saitou and Nei, 1987) based on 16S rRNA gene sequences of strains SA29-B^T^ and YU21-B in comparison with closely related species within *Steroidobacteraceae* and other *Nevskiales* in *Gammaproteobacteria*. The number of nodes indicate bootstrap values calculated based on a 1,000 resampled dataset. Bar, 0.01 substitutions per nucleotide position. Sequences of at least 1,400 nt were used for the calculation.

Strains SA29-A and YU21-A showed 99.4 and 99.6% similarities to *Rhizobium* sp. SEMIA 6437 (FJ025118) and *Bosea* sp. AS-1 (CP022372), respectively. Both bacteria are members of the family *Rhizobiales*. Strains SA29-B^T^ and YU21-B showed 99.3 and 99.2% similarities to *S. agariperforans* KA5-B^T^ (AB174844), which was the most closely related species. The phylogenetic tree constructed by the neighbor-joining algorithm indicated that strains SA29-B^T^ and YU21-B were closely affiliated with the species belonging to the genera *Steroidobacter* and *Povalibacter* (Fig. 1). Similarities in strains SA29-B^T^ and YU21-B were 97.3 and 97.3% for *S. soli* JW-3^T^ (MK311353), 96.8 and 96.9% for *S. denitrificans* FS^T^ (EF605262), 97.2 and 97.2% for *P. uvarum* Zumi 37^T^ (AB548216), 98.9 and 98.8% for “*S. cummioxidans*” 35Y (NZ_LSRW01000126), and 97.6 and 97.6% for “*S. flavus*” CPCC 100154 (KU195414), respectively. The phylogenetic analysis, constructed with maximum-parsimony and maximum-likelihood algorithms, also showed similar trees (available in supplementary material in Fig. S2). Based on their phylogenetic positions, strains SA29-B^T^ and YU21-B were suggested to be members of the genus *Steroidobacter*.

### Polysaccharide-degrading ability

Since strain KA5-B^T^ exhibited the ability to degrade not only agar, but also various polysaccharides, degrading abilities were compared among three strains. Degrading abilities were confirmed using staining methods based on the appearance of a clear zone around each bacterial colony. As shown in Table 1, CM-cellulose, dextrin, and wheat arabinoxylan were commonly degraded. On the other hand, different degrading abilities were observed for debranched arabinan, larch arabinogalactan, polygalacturonic acid, and pectic galactan; strains SA29-B^T^ and YU21-B degraded the first three polysaccharides, while strain KA5-B^T^ degraded the latter. Strain YU21-B also degraded chitin, xylan, arabinan, galactomannan, and xyloglucan. These differences in polysaccharide-degrading abilities indicated that SA29-B^T^ and YU21-B were different species from KA5-B^T^ regardless of whether SA29-B^T^ and YU21-B were the same or different species.

### Whole-genome sequencing and ANI analysis

The whole-genome sequencing of strains SA29-B^T^ and YU21-B was performed using GCM. The assembled genomes of strains SA29-B^T^ and YU21-B were 9.05 and 3.85‍ ‍Mbp with 29 and 10 scaffolds, respectively. N50 values and sequence depth coverage were 763,140 and 2,004,913 bp and 143 and 123 for strains SA29-B^T^ and YU21-B, respectively. Strains SA29-B^T^ and YU21-B had genome sizes of 8,010,349 and 8,036,638 bp and genomic DNA G+C contents (mol%) were 62.3 and 62.2 mol%, containing 7,032 and 6,891 genes, respectively.

According to the type strain genome database on GCM, genome sizes and all the genes of closely related species were 7,651,871 bp and 6,620 for *S. agariperforans* KA5-B^T^, 9,374,132 bp and 7,629 for *S. soli* JW-3^T^, and 3,467,246 bp and 2,960 for *S. denitrificans* FS^T^. The G+C contents of both strains were within the range of type strains in the family *Steroidobacteraceae* of 61.9 to 62.6 mol%.

ANI is a widely used *in silico* tool as an overall genome-related index (OGRI) to confirm whether a strain belongs to a known species by calculating relatedness between the genome sequences of the strain and type strain of a species (Chun *et al.*, 2018). The proposed and generally accepted species boundary for ANI is 95–96%. Chun *et al.* (2018) previously reported that a combination of 16S rRNA gene similarity and OGRI was available in a systematic process to identify and recognize new species. In the case of the 16S rRNA gene showing ≥98.7%, higher than the previously accepted 97%, against that of the closely related type strain, it may be regarded as a new species if the value is <95–96% when analyzed by ANI.

In the present study, strains SA29-B^T^ and YU21-B showed 99.3 and 99.2% similarities in 16S rRNA gene sequences against the most closely related strain KA5-B^T^. However, since the two strains were presumed to be different species based on their polysaccharide-degrading abilities, an ANI analysis was performed among strains SA29-B^T^, YU21-B, and KA5-B^T^. As shown in Table 2, the ANI value between strains SA29-B^T^ and YU21-B was 99.3%. The ANI values of SA29-B^T^ and YU21-B for KA5-B^T^ were 90.7 and 90.8%, respectively. This result indicated that strains SA29-B^T^ and YU21-B were the same species, while both strains were different species from *S. agariperforans* KA5-B^T^. These results suggested that strains SA29-B^T^ and YU21-B were novel species in the genus *Steroidobacter*.

### Ecological distribution

In order to investigate the ecological distribution of bacterial sequences affiliated with two novel strains and *S. agariperforans* KA5-B^T^, the rhizosphere soils from several types of plants were collected from Aomori prefecture to Okinawa prefecture in consideration of regional differences. Non-rhizosphere soils were also collected from the same fields in Ibaragi, Nagasaki, and Kagoshima prefectures.

**Fig. 2.**
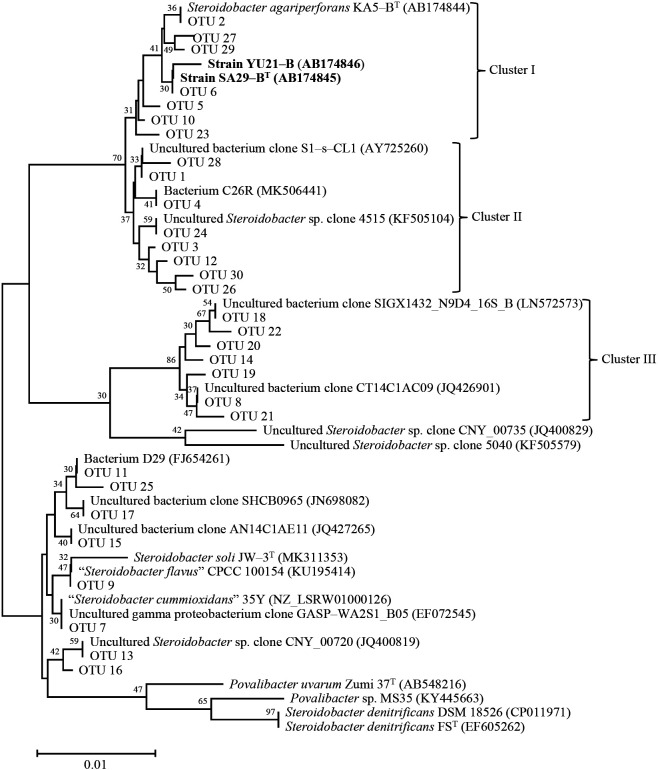
Neighbor-joining tree (Saitou and Nei, 1987) based on 16S rRNA gene sequences of strains SA29-B^T^ and YU21-B, *Steroidobacter* OTUs in rhizosphere and non-rhizosphere soils of respective plants with more than 1% relative abundance of any one of them, and closely related sequences belonging to genera *Steroidobacter* and *Povalibacter* in the family *Steroidobacteraceae*. Bar, 0.01 substitutions per nucleotide position.

Table 3 shows the results of the amplicon analysis of partial 16S rRNA genes amplified with KUSBf and KUSBr. The number of reads obtained was in the range of 16,593 to 38,262. OTUs that showed a percentage of 1% or higher in any of the soil samples were shown in the order of the highest total percentage. Thirty OTUs were identified, all of which were closely related to the genus *Steroidobacter*. However, not only OTUs closely affiliated with the three strains SA29-B^T^, YU21-B, and KA5-B^T^, but also the sequences closely related to *S. soli* JW-3^T^ (MK311353), “*S. flavus*” CPCC 100154 (KU195414), and “*S. cummioxidans*” 35Y (NZ_LSRW01000126) were detected for some OTUs (no. 7, 9, 11, 13, 15, 17, and 14) due to the similarity of sequences in the primer-annealing sites (Fig. 2).

Among the 30 OTUs in Table 3, OTU 6 and OTU 2 were identical sequences to SA29-B^T^ and KA5-B^T^, respectively. However, an OTU identical to YU21-B was not found in any sequences obtained by the amplicon analysis, suggesting that strain YU21-B is a bacterium with a limited habitat. OTU 2 had the second highest percentage among all OTUs, accounting for more than 10% in the rhizospheres of wax gourd and sweet potato in Okinawa prefecture. Although OTU 6 was not as high as OTU 2, it was one of the highest OTUs in all samples, and present in some of the potato rhizospheres of Ibaraki prefecture, the tomato rhizosphere of Kagoshima prefecture, and the wax gourd and sweet potato rhizospheres in Okinawa prefecture. Variations in percentages in the different regions, plants, and rhizosphere and non-rhizosphere soils may be attributed to the species composition of *Rhizobiales* bacteria. Although SA29-B^T^, YU21-B, and KA5-B^T^ require the metabolites of *Rhizobiales* bacteria for regular growth, the response of each strain to these metabolites may differ according to the species composition of *Rhizobiales* bacteria. In the present study, we only evaluated ecological distributions based on an OTU sequence identical to the strains. Further studies are needed to investigate relevance to the species composition of *Rhizobiales* bacteria.

On the other hand, the NGS analysis detected other OTU sequences closely related to the three strains, except for OTUs 6 and 2 (Fig. 2). These OTUs formed three clusters, consisting of clusters I, II, and III. Clusters I, II, and III contained OTUs 5, 10, 23, 27, and 28, OTUs 1, 3, 4, 12, 26, 28, and 30, and OTUs 8, 14, 18, 18, 20, 21, and 22, respectively. This variety of OTUs suggested that bacteria affiliated with the three strains were widely distributed throughout rhizosphere and non-rhizosphere soils.

In addition, three strains in cluster I and bacterium S26R (MK506441) (Rebollar *et al.*, 2019) in cluster II were the bacteria that had not been previously isolated. There were no isolates in cluster III; only uncultured bacterium clones SIGX1432_N9D4_16S_B (LN572573) and CT14C1AC09 (JQ426901) were obtained. Based on the phylogenetic distance between the two strains and *S. agariperforans* KA5-B^T^, OTUs in cluster I, for example 5, 10 and 23, and all OTUs 1, 3, 4, 8, 12, 14, 18, 19, 20, 21, 22, 24, 26, 28, and 30 in clusters II and III were displayed at some distances, suggesting that a novel species is present in these clusters.

### Morphological and physiological characteristics

To morphologically and physiologically characterize strains SA29-B^T^ and YU21-B, standard tests were performed, including colony features, Gram staining, motility, cell morphology, spore formation, cell size, respiratory, catalase and cytochrome oxidase activities, and growth ranges at different temperatures, pH, and NaCl concentrations (Table 4).

**Fig. 3.**
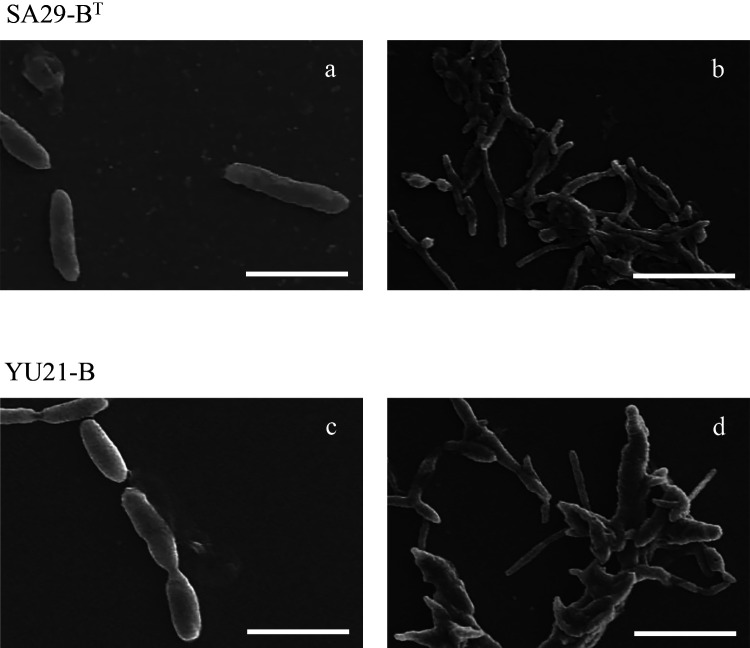
Scanning electron micrograph of strains SA29-B^T^ and YU21-B. Bars, (a) and (c) 2‍ ‍μm, and (b) and (d) 4‍ ‍μm.

The colonies of both strains were smooth, circular, and pale-yellow in color. The cells of both strains were Gram-negative. Strains SA29-B^T^ and YU21-B were non-motile, rod-shaped, and non-spore-forming, while strain KA-5B^T^ was straight to slightly curved rods. Cell sizes were a width of 0.4–0.6‍ ‍μm and length of 1.5–2.5‍ ‍μm for strain SA29-B^T^, and a width of 0.4–0.7‍ ‍μm and length of 1.3–2.0‍ ‍μm for strain YU21-B (Fig. 3a and c). Both strains were mostly included in a fibrous polysaccharide matrix (Fig. 3b and d), similar to strain KA5-B^T^.

Physiological analyses revealed that strains SA29-B^T^ and YU21-B did not grow under anaerobic conditions and had positive reactions for catalase and cytochrome oxidase activities. Visible colonies were observed between 15–40°C for both strains with optimum growth temperatures of 35°C for strain SA29-B^T^ and 33–35°C for strain YU21-B. Both strains grew between pH 6.0–12.0, with optimum growth at pH 7.0–7.5 for strain SA29-B^T^ and at pH 7.0 for strain YU21-B, and at NaCl concentrations of 0–2% for strain SA29-B^T^ and of 0–3% for strain YU21-B, with optimum growth at 1%. In comparisons with strain KA5-B^T^, the upper limits of the growth temperature and pH at which KA5-B^T^ grows were 37°C and pH 9.0, with a difference in growth at 40°C and pH 12.

### Chemotaxonomic characteristics

Whole fatty acids and the main quinones were analyzed after the extraction and purification of the respective targeted matter from the cells of strains SA29-B^T^ and YU21-B (Table 4). The dominant fatty acids were C_16:0_ (17.5 and 11.7% for SA29-B^T^ and YU21-B, respectively), iso-C_15:0_ (15.5 and 16.3% for SA29-B^T^ and YU21-B, respectively), iso-C_17:0_ (19.0 and 18.3% for SA29-B^T^ and YU21-B, respectively), summed features 3 (15.9 and 12.3% for SA29-B^T^ and YU21-B, respectively), and summed features 9 (11.3% for YU21-B). Summed features 3 are composed of C_16:1_ω7c and C_16:1_ω6c, and Summed features 9 of iso-C_17:1_ω9c and C_16:0_ 10-methyl I. The fatty acids present at moderate amounts were C_12:0_ (5.9 and 6.0% for SA29-B^T^ and YU21-B, respectively), iso-C_16:0_ (6.7 and 9.8% for SA29-B^T^ and YU21-B, respectively), and summed features 9 (7.6% for SA29-B^T^). The whole-cell fatty acid profiles of both strains and the species in the family *Steroidobacteraceae* are shown in supplementary Table S1. C_16:0_ and iso-C_17:0_, which were detected as major fatty acids, were present at moderate amounts in strain KA5-B^T^. iso-C_17:0_ was detected at a small amount, less than 5% or below the detection limit, in the other closest relatives. In addition, iso-C_11:0_ was specific to strains SA29-B^T^, YU21-B, and KA5-B^T^, while C_18:0_ and iso-C_18:0_ were specific to strains SA29-B^T^ and YU21-B.

An analysis of the respiratory quinone composition indicated that Q-8 was the main quinone for strains SA29-B^T^ (98.5%) and YU21-B (98.3%). The other quinone was Q-9 for both strains. Q-8 was commonly detected in species not only in the family *Steroidobacteraceae*, but also in the order *Nevskiales* (Suyama *et al.*, 1993; Gutierrez *et al.*, 2012; Liu *et al.*, 2019; Wu and Ma, 2019).

### Substrate utilization

As shown in Table 5, the following substrates in Biology GN2 were utilization-positive for both strains; L-alaninamide, α-cyclodextrin, dextrin, D-galactose, gentiobiose, D-glucose, glycyl-L-glutamate, β-hydroxy butyrate, α-D-lactose, L-leucine, maltose, L-phenylalanine, L-proline, L-rhamnose, and tween 40. D,L-lactate was weakly positive for both strains. N-acetyl-D-galactosamine, γ-aminobutyrate, D-cellobiose, L-glutamate, L-histidine, methyl β-D-glucoside, D-trehalose, turanose, and tween 80 were positive, and N-acetyl-D-glucosamine, α-ketoglutarate, and succinamate were weakly positive for strain SA29-B^T^. On the other hand, hydroxy-L-proline and D,L-lactate were weakly positive for strain YU21-B.

D-raffinose, the pyruvate methyl ester, and sucrose were positive, and D-fructose, D-glucose 6-phosphate, L-alanine, and lactulose were weakly positive for *S. agariperforans* KA5-B^T^, while these substrates were negative for both strains. The leftover substrates, including acetate and citrate, were also negative for both strains.

Therefore, strains SA29-B^T^ and YU21-B were considered to be the same species and showed suitable characteristics for classification as novel species in the genus *Steroidobacter* of the family *Steroidobacteraceae*. The bacterial sequences affiliated with the two strains were widely distributed in the rhizosphere, but varied in plant- and region-dependent manners.

Based on polyphasic approaches, we propose a novel species, for which the name *Steroidobacter agaridevorans* sp. nov. is proposed. The type strain is SA29-B^T^.

### Description of *Steroidobacter agaridevorans sp. nov.*

*Steroidobacter agaridevorans* (a.ga.ri.de.vo'rans. Malayan n. *agar* agar [algal polysaccharide]; N.L. neut. n. *agarum* agar; L. pres. part. adj. devorans consuming, devouring; N.L. part. adj. *agaridevorans* agar-devouring).

Its colonies are smooth and pale yellow in color. Cells are Gram-negative, non-motile, rod-shaped with a width of 0.4–0.6‍ ‍μm and length of 1.5–2.5‍ ‍μm (type strain), and non-spore-forming. Most cells are included in a fibrous polysaccharide matrix. It does not grow under anaerobic conditions. Cytochrome oxidase and catalase activities are positive. It exhibits the ability to degrade agar. Diffusible metabolites from *Rhizobiales* bacteria are required for regular growth. The growth of the type strain occurs at 15–40°C (optimum 35°C), pH 6.0–12.0 (optimum pH 7.0–7.5), and NaCl concentrations of up to 2% (w/v) (optimum 1%). The dominant whole cell fatty acids are C_16:0_, iso-C_15:0_, iso-C_17:0_, and summed features 3. The main quinone is Q-8. The genomic DNA G+C content of the type strain is 62.3 mol%. Substrate utilization of the type strain by Biolog GN2 is positive for N-acetyl-D-galactosamine, L-alaninamide, γ-aminobutyrate, D-cellobiose, α-cyclodextrin, dextrin, D-galactose, gentiobiose, D-glucose, L-glutamate, glycyl-L-glutamate, L-histidine, β-hydroxy butyrate, α-D-lactose, L-leucine, maltose, methyl β-D-glucoside, L-phenylalanine, L-proline, L-rhamnose, D-trehalose, turanose, tween 40, and tween 80, weakly positive for N-Acetyl-D-glucosamine, α-Ketoglutarate, D,L-Lactate, and Succinamate, and negative for acetate and citrate. The type strain is *Steroidobacter agaridevorans* SA29-B^T^ (JCM 333368^T^=KCTC 72223^T^), which was isolated from a rhizosphere soil sample.

## Citation

Ikenaga, M., Kataoka, M., Yin, X., Murouchi, A., and Sakai, M. (2021) Characterization and Distribution of Agar-degrading *Steroidobacter agaridevorans* sp. nov., Isolated from Rhizosphere Soils. *Microbes Environ ***36**: ME20136.

https://doi.org/10.1264/jsme2.ME20136

## Supplementary Material

Supplementary Material

## Figures and Tables

**Table 1.** Polysaccharide-degrading abilities of strains SA29-B^T^, YU21-B, and the most closely related *Steroidobacter agariperforans* strain KA5-B^T^ (Sakai *et al.*, 2014).

	SA29-B^T^	YU21-B	KA5-B^T^
Agar	+	+	+
Arabinan	–	+	–
Chitin	–	+	–
CM-cellulose	+	+	+
Debranched arabinan	+	+	–
Dextrin	+	+	+
Galactomannan	–	+	–
Larch arabinogalactan	+	+	–
Pectic galactan	–	–	+
Polygalacturonic acid	+	+	–
Starch	–	+	+
Wheat arabinan	+	+	+
Xylan	–	+	–
Xyloglucan	–	+	–

Symbols + and – indicate positive and negative, respectively.

**Table 2.** Values of Average Nucleotide Identity (ANI) between two novel strains (SA29-B^T^ and YU21-B) and the most closely related *Steroidobacter agariperforans* strain KA5-B^T^ (Sakai *et al.*, 2014)

	SA29-B^T^	YU21-B
YU21-B	99.3	—
KA5-B^T^	90.7	90.8

GeneBank accession numbers for genomic information on the strains used in this analysis were GCA_009932555.1, GCA_009932515.1, and GCA_004138335.1 for SA29-B^T^, YU21-B, and KA5-B^T^, respectively.

**Table 3.** Percentage of *Steroidobacter* OTUs in rhizosphere and non-rhizosphere soils of respective plants with an abundance of more than 1% of any one of them.

OTUs	Aomori		Ibaragi				Nagasaki			Kagoshima				Okinawa		
	Garlic		Non-rhizo	Onion	Potato		Non-rhizo	Potato		Non-rhizo	Soybean	Tomato		Wax gourd	Sugarcane	Sweet potato
1	2.69		5.19	3.39	1.68		4.53	27.16		0.97	4.54	12.26		0.82	0.63	1.03
2	0.01		1.46	0	6.46		0.01	0		8.16	3.76	0.51		14.74	0.44	18.70
3	3.51		14.46	26.06	0.08		1.52	1.46		0	0.01	0.05		0.05	0.72	0.01
4	0.71		2.22	0.33	2.13		1.51	0.47		6.47	5.07	3.94		4.69	0.97	1.60
5	0.01		0.24	0.01	3.01		0.04	0.01		11.36	1.20	0.64		2.67	0.39	1.73
6	0.01		0.26	0	4.45		0.03	0.08		0.40	0.65	3.25		2.30	0.02	3.86
7	0.72		0.29	0.18	0.08		3.66	0.92		0.20	3.02	4.46		0.03	0.16	0.07
8	0		0	0	0.14		0	0		0.22	0.32	0		0.11	11.48	0.03
9	1.04		0.24	0.12	0.13		2.87	0.10		0.56	3.45	3.09		0.24	0.29	0.17
10	0.02		0.50	0.04	1.76		0.03	0.28		0.90	1.11	1.65		0.57	0.26	0.81
11	2.77		0.06	0	0.16		1.58	0.02		0.20	0.94	0.58		0.35	0.41	0.30
12	2.18		1.80	0.84	0.10		1.19	0.09		0.01	0	0.03		0.04	0.06	0
13	0.90		0.01	0	0.01		3.14	1.39		0	0	0.04		0	0.01	0.01
14	0		0	0	0.61		0	0		0.45	2.33	0		0.92	0.62	0.27
15	1.38		0.03	0	0.04		2.26	0.31		0.03	0.26	0.67		0.04	0.06	0.05
16	0.36		0.14	0.08	0.01		2.53	1.73		0	0.02	0.16		0	0.01	0
17	0.05		0.04	0	0.41		0.01	0.00		0.60	1.27	0.44		0.59	0.12	1.50
18	0		0	0	1.34		0	0		0.28	0.37	0		1.33	0.20	1.04
19	0		0	0	0.12		0	0		0.13	0.08	0		0.06	4.10	0.02
20	0		0	0	1.43		0	0		0.49	0.53	0		0.45	0.26	0.19
21	0		0	0	0.01		0	0		0.01	0	0		0	2.49	0
22	0		0	0	0.61		0	0		0.30	0	0		1.18	0	0
23	0.04		1.50	0.20	0.06		0.02	0.02		0.01	0	0		0.03	0.18	0
24	0		0.03	0	1.26		0	0		0	0	0		0.57	0	0
25	1.69		0.01	0.01	0		0	0		0.01	0	0		0	0	0.01
26	1.17		0.37	0.09	0		0	0		0	0	0		0	0.01	0
27	0		0.00	0	1.04		0	0		0	0	0		0.59	0	0
28	0.05		0.01	0.01	0.01		1.30	0.12		0.01	0.02	0.03		0	0	0
29	0		0	0	1.14		0	0		0	0	0		0.40	0	0
30	1.10		0.09	0	0		0	0		0	0	0		0	0	0

More than 20% for purple, more than 15 to 20% for red, more than 10 to 15% for orange, more than 5 to 10% for yellow, more than 3 to 5% for light green, more than 1 to 3% for light blue, and more than 0 to 1% for light grey. Non-rhizo means non-rhizosphere soil.

**Table 4.** Phenotypic and chemotaxonomic characteristics of strains SA29-B^T^ and YU21-B in comparison with the closely related species belonging to genera *Steroidobacter* and *Povalibacter* in the family *Steroidobacteraceae*.

	1	2	3	4	5	6	7	8
Colony color	pale yellow	pale yellow	pale yellow	pale yellow	yellow-brown	yellow-orange	light yellow, then turns to yellow	light brown
Motility	non-motile	non-motile	non-motile	motile	motile by a single polar flagellum	motile by 1 (or 2) polar flagellum	motile by a single polar flagellum	motile by a single polar flagellum
Cell morphology	Rod	rod	straight to slightly curved rod	straight to slightly curved rod	slightly curved rod	rod	rod	rod
Cell size width by length (μm)	0.4–0.6 by 1.5–2.5	0.4–0.7 by 1.3–2.0	0.4–0.6 by 1.0–2.1	0.3–0.5 by 1.0–3.1	0.3–0.5 by 0.6–1.6	0.4–0.6 by 2.0–5.0, occasionally longer (10‍ ‍μm)	0.6–0.8 by 1.5–1.8	0.3–0.5 by 1.0–3.0
Anaerobic growth	–	–	–	–	–	+	–	–
Catalase	+	+	+	+	+	–	+	+
Cytochrome oxidase	+	+	+	+	+	+	–	+
Growth range at different temperatures (optimum) (°C)	15–40 (35)	15–40 (33–35)	15–37 (30)	16–37 (30)	20–38 (28–30)	25–41 (ND)	20–37 (28–32)	13–33 (30)
Growth range at different pH (optimum)	6.0–12.0 (7.0–7.5)	6.0–12.0 (7.0)	4.5–9.0 (6.0–8.0)	6.0–9.0 (7.0)	6.1–7.8 (7.0)	ND (ND)	6.0–8.0 (7.0–7.5)	6.5–9.0 (7.5 or 7.0)
Growth range at different NaCl concentrations (optimum) (%)	0–2.0 (1.0)	0–3.0 (1.0)	0–2.0 (ND)	0–1.0 (0.5)	ND (0.1)	ND (ND)	0–3.0 (ND)	0–0.5 (0.1)
Growth at 40°C	+	+	–	–	–	+	–	–
Growth at pH 12	+	+	–	–	–	ND	–	–
Utilization of agar as the C source	+	+	+	ND	–^a^	–	ND	ND
Growth on solid plates	+	+	+	+	–	+	+	+
Dominant whole-cell fatty acid (≥10%)	C_16:0_, iso-C_15:0_, iso-C_17:0_, Sum3	C_16:0_, iso-C_15:0_, iso-C_17:0_, Sum3, Sum9	iso-C_15:0_, Sum3, Sum9	C_12:0_, isoC_16:0_, Sum3	C_15:0_, C_17:1_ω8c	ND	C_16:0_, Sum3	iso-C_15:0_, iso-C_16:0_, Sum3
Genomic G+C content (mol%)	62.3	62.2	62.0	62.6	61.9	60.9	64.4	64.2

Taxa: 1 strain SA29-B^T^; 2 strain YU21-B; 3, *Steroidobacter agariperforans* KA5-B^T^ (Suyama *et al.*, 1993); 4, *Steroidobacter soli* JW-3^T^ (Huang *et al.*, 2019); 5, *Steroidobacter denitrificans* FS^T^ (Fahrbach *et al.*, 2008); 6, “*Steroidobacter cummioxidans*” 35Y (Sharma *et al.*, 2018); 7, “*Steroidobacter flavus*” CPCC 100154 (Gong *et al.*, 2016); 8, *Povalibacter uvarum* Zumi 37^T^ (Nogi *et al.*, 2014). Sum3, summed feature 3, is composed of C16:1ω7c and C16:1ω6c. Sum9, summed feature 9, is composed of iso-C17:1ω9c and C16:0 10-methyl I. All strains are Gram-negative, non-spore-forming and containing Q-8 as the main quinone. +, positive; ±, weakly positive; –, negative; ND, not determined or no data available. The data shown in superscript a were obtained from Sharma *et al.* (2018).

**Table 5.** Substrate utilization by strains SA29-B^T^ and YU21-B in comparison with the most closely related *Steroidobacter agariperforans* strain KA5-B^T^.

Substrates	1	2	3
L-alaninamide	+	+	±
α-cyclodextrin	+	+	±
Dextrin	+	+	+
D-galactose	+	+	+
Gentiobiose	+	+	+
D-glucose	+	+	+
Glycyl-L-glutamate	+	+	–
β-hydroxy butyrate	+	+	±
α-D-lactose	+	+	+
L-leucine	+	+	–
Maltose	+	+	±
L-phenylalanine	+	+	–
L-proline	+	+	±
L-rhamnose	+	+	+
Tween 40	+	+	+
D,L-lactate	±	±	±
N-acetyl-D-galactosamine	+	–	–
γ-aminobutyrate	+	–	–
D-cellobiose	+	–	+
L-glutamate	+	–	±
L-histidine	+	–	–
Methyl β-D-glucoside	+	–	+
D-trehalose	+	–	+
Turanose	+	–	+
Tween 80	+	–	–
N-acetyl-D-glucosamine	±	–	–
α-ketoglutarate	±	–	+
Succinamate	±	–	–
Hydroxy-L-proline	–	±	±
D,L-lactate	–	±	±
D-raffinose	–	–	+
Pyruvate methyl ester	–	–	+
Sucrose	–	–	+
D-fructose	–	–	±
D-glucose 6-phosphate	–	–	±
L-alanine	–	–	±
Lactulose	–	–	±
Acetate	–	–	–
Citrate	–	–	–

Taxa: 1 strain SA29-B^T^; 2 strain YU21-B; 3, *Steroidobacter agariperforans* KA5-B^T^ (Sakai *et al.*, 2014). Symbols +, ±, and – indicate positive, weakly positive, and negative substrate utilization, respectively. The leftover substrates in Biolog GN2 were utilization negative among the three strains.
