# Characterization and Distribution of Agar-degrading *Steroidobacter agaridevorans* sp. nov., Isolated from Rhizosphere Soils

**DOI:** 10.1264/jsme2.ME20136e

**Published:** 2021-03-23

**Authors:** Makoto Ikenaga, Machi Kataoka, Xuan Yin, Aya Murouchi, Masao Sakai

**Affiliations:** 1 Research Field in Agriculture, Agriculture Fisheries and Veterinary Medicine Area, Kagoshima University, 1–21–24, Korimoto, Kagoshima, 890–0065, Japan; 2 The United Graduate School of Agricultural Sciences, Kagoshima University, 1–21–24, Korimoto, Kagoshima, 890–0065, Japan; 3 Faculty of Agriculture, Kagoshima University, 1–21–24, Korimoto, Kagoshima, 890–0065, Japan; 4 Graduate School of Agriculture, Kagoshima University, 1–21–24, Korimoto, Kagoshima, 890–0065, Japan

Vol. 36, No. 1, ME20136, 2021

 

p.1 Abstract

Incorrect: JCM 333368^T^

Correct: JCM 33368^T^

 

Incorrect: JCM 333367

Correct: JCM 33367

 

p. 9 right column

Incorrect: JCM 333368^T^

Correct: JCM 33368^T^

## Description of *Steroidobacter agaridevorans sp. nov.*

*Steroidobacter agaridevorans* (a.ga.ri.de.vo'rans. Malayan n. *agar* agar [algal polysaccharide]; N.L. neut. n. *agarum* agar; L. pres. part. adj. devorans consuming, devouring; N.L. part. adj. *agaridevorans* agar-devouring).

Its colonies are smooth and pale yellow in color. Cells are Gram-negative, non-motile, rod-shaped with a width of 0.4–0.6‍ ‍μm and length of 1.5–2.5‍ ‍μm (type strain), and non-spore-forming. Most cells are included in a fibrous polysaccharide matrix. It does not grow under anaerobic conditions. Cytochrome oxidase and catalase activities are positive. It exhibits the ability to degrade agar. Diffusible metabolites from *Rhizobiales* bacteria are required for regular growth. The growth of the type strain occurs at 15–40°C (optimum 35°C), pH 6.0–12.0 (optimum pH 7.0–7.5), and NaCl concentrations of up to 2% (w/v) (optimum 1%). The dominant whole cell fatty acids are C_16:0_, iso-C_15:0_, iso-C_17:0_, and summed features 3. The main quinone is Q-8. The genomic DNA G+C content of the type strain is 62.3 mol%. Substrate utilization of the type strain by Biolog GN2 is positive for N-acetyl-D-galactosamine, L-alaninamide, γ-aminobutyrate, D-cellobiose, α-cyclodextrin, dextrin, D-galactose, gentiobiose, D-glucose, L-glutamate, glycyl-L-glutamate, L-histidine, β-hydroxy butyrate, α-D-lactose, L-leucine, maltose, methyl β-D-glucoside, L-phenylalanine, L-proline, L-rhamnose, D-trehalose, turanose, tween 40, and tween 80, weakly positive for N-Acetyl-D-glucosamine, α-Ketoglutarate, D,L-Lactate, and Succinamate, and negative for acetate and citrate. The type strain is *Steroidobacter agaridevorans* SA29-B^T^ (JCM 33368^T^=KCTC 72223^T^), which was isolated from a rhizosphere soil sample.

 

