# Supplementary material for: Characterization and Distribution of Agar-degrading Steroidobacter agaridevorans sp. nov., Isolated from Rhizosphere Soils
Source: Microbes Environ. 2021 Mar 13;36(1):ME20136. doi: 10.1264/jsme2.ME20136 (PMC7966939; doi:10.1264/jsme2.ME20136)
Supplement: Supplementary file 1 — Supplementary Material [file 36_20136_s1.pdf]

Supplementary Table S1. Whole cell fatty acids composition of strains SA29-B^T and YU21-B in comparison with the closely related species belonged to genus *Steroidobacter* and *Povalibacter* in the family of *Steroidobacteraceae*.

	1	2	3	4	5		6	7
					testosterone	heptanoate		
$C_{9:0}$					0.6	0.8	2.3	
$C_{10:0}$				TR	1.3	0.2		
$C_{11:0}$					2.7	4.2		5.9
$C_{12:0}$	5.9	6.0	4.7	10.8	1.8	0.2	6.4	2.3
$C_{13:0}$					4.2	6.6		
$C_{14:0}$	0.4	0.3		TR	0.9		1.4	1.1
$C_{15:0}$					16.5	19.5		
$C_{16:0}$	17.5	11.7	8.7	2.5	7.0	0.7	29.6	4.7
$C_{17:0}$	0.6	0.4			6.2	5.4		
$C_{18:0}$	0.5	0.3						
$C_{12:0}$ 2-OH	1.2	1.3	2.7	1.7			7.1	
$C_{11:0}$ 3-OH					1.2	1.6		
$C_{12:0}$ 3-OH	3.1	3.6	4.6	1.9	1.3	0.2	4.3	1.3
iso-$C_{16:0}$ H					0.8	1.5		
iso-$C_{11:0}$	1.9	2.4	3.9					
iso-$C_{12:0}$	0.3	0.5						1.6
iso-$C_{13:0}$	1.5	2.1						2.5
iso-$C_{14:0}$			0.3					1.3
iso-$C_{15:0}$	15.5	16.3	18.1	8.4				18.7
iso-$C_{16:0}$	6.7	9.8	5.6	16.7			1.1	13.0
iso-$C_{17:0}$	19.0	18.3	7.9	1.6				1.4
iso-$C_{18:0}$	0.2	0.3						
iso-$C_{13:0}$ 3-OH	1.3	1.8						1.2
$C_{15:1}\omega6c$						0.3		
$C_{15:1}\omega8c$					2.5	4.0		
$C_{16:1}\omega7c$								40
$C_{16:1}\omega9c$					0.9			
$C_{17:1}\omega6c$					3.0	3.9		
$C_{17:1}\omega8c$					27.1	40.6		
iso-$C_{17:1}\omega9c$								
iso-$C_{17:1}\omega10c$								5.1
$C_{18:1}\omega7c$					4.2	0.5		
$C_{16:0}$ 10-methyl I					7.0	1.5		
$C_{17:0}$ 10-methyl I					2.4	2.7		
Summed features 1						0.6		
Summed features 3	15.9	12.3	32.1	35.5	8.3	0.7	46.1	
Summed features 9	7.6	11.3	11.8	9.2				

Taxa: 1, strain SA29-B^T; 2, strain YU21-B; 3, *Steroidobacter agariperforans* KA5-B^T (Sakai *et al*., 2014); 4, *Steroidobacter soli* JW-3^T (Huang *et al*., 2019); 5, *Steroidobacter denitrificans* FS^T (Fahrbach *et al*., 2008); 6, "*Steroidobacter flavus*" CPCC 100154 (Gong *et al*., 2016); 7, *Povalibacter uvarum* Zumi 37^T (Nogi *et al*., 2014). Summed features 1 consists of iso-$C_{15:1}$ H and/or $C_{13:0}$ 3-OH, summed features 3 consists of $C_{16:1}\omega7c$ and/or $C_{16:1}\omega6c$ except that of strain *S. denitrificans* FS^T, and summed features 9 consists of iso-$C_{17:1}\omega9c$ and/or $C_{16:0}$ 10-methyl I. Summed features 3 of strain FS^T consists of $C_{16:1}\omega7c$ and/or iso-$C_{15:0}$ 2-OH. TR indicates an amount of less than 1%. The fatty acid of strain 35Y^T had not been determined in the reference Sharma *et al*., (2018).

Supplementary Fig. S1. Design of primers (KUSBf and KUSBr) specific to the three strains, SA29-B^{T}, YU21-B and KA5-B^{T}.

Escherichia coli position on 16S rRNA gene	135 140 145 150 155 160 165 250 255 260 450 455 460 465
**KUSBf primer**	CTGTTAGTGGGGGACAACCAA
**KUSBr primer**	TGCGACCTAACACGTCGC
**Strain SA29-B^{T} (AB174845)**	CTGTTAGTGGGGGACAACCAACCGAAAGGTTGG--TGGTTGGTAGGGTAATG--AAGCTGCGACCTAACACGTCGC
**Strain YU21-B (AB174846)**	CTGTTAGTGGGGGACAACCAACCGAAAGGTTGG--TAGTTGGTAGGGTAATG--AAGCTGCGACCTAACACGTCGC
***Steroidobacter agariperforans* KA5–B^{T} (AB174844)**	CTGTTAGTGGGGGACAACCAACCGAAAGGTTGG--TAGTTGGTAGGGTAACG--AAGCTGCGACCTAACACGTCGC
Steroidobacteraceae	
Steroidobacter sp. JC2953 (KF595153)	CCTCTGGTGGGGAATAACCAGCCGAAAGGTTGG--TAGTTGGTGAGGTAACG--AAGTGCAGAGCTAACACCTCTG
Uncultured *Steroidobacter* sp. A483 (JF833541)	CCAATGGTGGGGAATAACCAGCCGAAAGGTTGG--TAGTTGGTAGGGTAACG--AAGTGCAGAGCTAATATCTCTG
Steroidobacter sp. JC2986 (KP185148)	CCTATGGTGGGGAATAACCAGCCGAAAGGTTGG--TAGTTGGTGAGGTAACG--ATGTGCAGGGTTAATACCTCTG
Uncultured *Steroidobacter* sp. A143 (JF833490)	CCAATGGTGGGGAATAACCAGCCGAAAGGTTGG--TAGTTGGTAGGGTAACG--ATGTTCACGGCTAATACCCGTG
Steroidobacter sp. Br1-25 (KY908253)	CCAGTAGTGGGGAATAACTAACCGAAAGGTTAG--TAGTTGGTGAGGTAACG--AAGCACAGGGCTAATACCTCTG
Uncultured *Steroidobacter* sp. A4-16 (KF469200)	CTAGTAGTGGGGAATAACCCGGCGAAAGCCGGG--TAGTTGGTAGGGTAACG--ATATGCCGACCTAATACGTCGG
"Steroidobacter cummioxidans" 35Y (NZ_LSRW01000126)	CTGTTAGTGGGGGACAACCCGGGGAAACTCGGG--TAGTTGGTAGGGTAATG--AAGTTGCGACCTAACACGTCGC
Uncultured Steroidobacter sp. b18-164 (JX576027)	CTGTTAGTGGGGGACAACCCGGGGAAACTCGGG--TAGTTGGTAGGGTAACG--AAGTTGCGACCTAACACGTCGC
Bacterium D29 (FJ654261)	CTGTTAGTGGGGGACAACCCGGGGAAACTCGGG--TAGTTGGTAGGGTAACG--AAGTTGCGACCTAACACGTTGC
"Steroidobacter flavus" CPCC 100154 (KU195414)	CTGTTAGTGGGGGACAACCCGGGGAAACTCGGG--TAGTTGGTAGGGTAACG--AAGTTGCGGTTTAATACATCGC
Steroidobacter soli JW-3^{T} (MK311353)	CTGTTAGTGGGGGACAACACGGGGAAACTCGTG--TAGTTGGTAGGGTAACG--AAGTTGCGGTTTAACACATCGC
Uncultured *Steroidobacter* sp. SBL03 (KM108708)	CTGTTAGTGGGGGACAACCCGGGGAAACTCGGG--TAGTTGGTAGGGTAACG--AAGCCTCGAGCTAACACCTCGG
Povalibacter uvarum Zumi 37^{T} (AB548216)	CTATTAGTGGGGGACAACCCGGGGAAACTCGGG--TAGTTGGTAGGGTAATG--AAGCCTCGAGTTAACACCTCGG
Uncultured *Steroidobacter* sp. De32 (HQ183826)	CTATTAGTGGGGGATAACTCGGGGAAACTCGAG--TAGTTGGTAGGGTAATG--AAGCCTCGAGTTAATATCTCGG
Steroidobacter denitrificans FS^{T} (EF605262)	CTATTAGTGGGGGATAACTCGGGGAAACTCGAG--TAGTTGGTAGGGTAATG--AAGCCTCGAGTGAATACCTCGG
Steroidobacter denitrificans DSM 18526 (CP011971)	CTATTAGTGGGGGATAACTCGGGGAAACTCGAG--TAGTTGGTAGGGTAATG--AAGCCTCGAGTGAATACCTCGG
Uncultured *Steroidobacter* sp. S244 (JN217087)	CTATCAGTGGGGGACAACCCGGGGAAACTCGGG--TAGTTGGTAGGGTAATG--AAGTCTCGACCTAATACGTCGG
Uncultured *Steroidobacter* sp. P1 (HE648175)	CTATCAGCGGGGGACAACCCGGGGAAACTCGGG--TAGTTGGCGGGGTAACG--ATCTCTTGCTCTAACACGGCAG
Uncultured *Steroidobacter* sp. P2 (HE648176)	CTATCAGCGGGGGACAACCCGGGGAAACCCGGG--TAGTTGGCGGGGTAACG--AACTCTCGACTTAACACGTCGA
**Other *Nevskiales***	
Solimonas soli DCY12^{T} (EF067861)	CTTAGAGCGGGGGATAGCCCAGGGAAACTTGGA--TAGTTGGTAGGGTAATG--AAAACGTGCTCTAACATAGCGC
Solimonas flumis HR-BB^{T} (MF682434)	CTTAGAGCGGGGGATAGCCCAGGGAAACTTGGA--TAGTTGGTGAGGTAATG--AAAACTCGGACTAACATTTCGA
Solimonas variicoloris MN28^{T} (NR_042175)	CTCAGAGCGGGGGATAGCCCAGGGAAACTTGGA--TAGTTGGTAGGGTAAAG--AAAACTCGGTCTAATACATCGA
Solimonas terrae KIS83-12^{T} (NR_125692)	CTTAGAGCGGGGGATAGCCCAGGGAAACTTGGA--TAGTTGGCGGGGTAATA--AAAATTTCACCTAATACGTGAA
Solimonas aquatica NAA16^{T} (NR_108189)	CTTTAGGTGGGGGATAGCCCAGGGAAACTTGGA--TAGTTGGTAGGGTAATG--AAAGTCGGAGCTAATATCTCCG
Fontimonas thermophila HA-01^{T} (NR_109471)	CTCAGAGTGGGGGATAGCCCGGGGAAACCCGGA--TAGTTGGTGGGGTAATG--AAAGCTCGACCTAATACGCCGA
Sinimarinibacterium flocculans NH6-24^{T} (HQ875491)	CTCAGAGTGGGGGATAGCCCGGGGAAACTCGGA--TAGTTGGCGGGGTAATG--AAAGCTCAAGCTAATATCTTGA
Nevskia lacus Seoho-38^{T} (NR_164939)	CTTTAAGTGGGGGATAACCCGGGGAAACCCGGA--TAGTTGGTGAGGTAATG--AAAGCTTCACCTAATACGTGAA
Nevskia soli GR15-1^{T} (NR_044135)	CCTTAAGTGGGGGATAACCCGGGGAAACCCGGA--TAGTTGGTGAGGTAATG--AAAATTCCACCTAATACGTGGA
Nevskia ramosa DSM 11499^{T} (NZ_ATVI01000012)	CTTTAAGTGGGGGATAACCCGGGGAAACCCGGA--TTGTTGGTGAGGTAATG--AAAACTTCAATTAATACTTGAA
Nevskia persphonica G6M-30^{T} (NR_109617)	CCTTAAGTGGGGGATAACCCGGGGAAACCCGGA--TAGTTGGTGAGGTAATG--AAAGCTTCAGCTAACATCTGAA
Nevskia aquatilis F2-63^{T} (NR_109616)	CCTTAAGTGGGGGATAGTCGGGGGAAACTCCGT--TAGTTGGTGAGGTAACG--AAAACTTTGGCTAACATCCAAA
Alkanibacter difficilis MN154.3^{T} (NR_042077)	CCTTGAGTGGGGGATAGCCCGGGGAAACTCGGA--TAGTTGGTAGGGTAAAG--AAAGCTTTGGTGAATATCCAAA
Hydrocarboniphaga daqingensis B2-9^{T} (NR_116271)	CCTTAAGTGGGGGATAACCCGGGGAAACCCGGA--TTGTTGGTGAGGTAATG--AAAACTTTCTCTAATACAGGAA
Polycyclovorans algicola TG408^{T} (NR_116560)	CCGAGAGTGGGGGATAACCCAGGGAAACTTGGA--TTGTTGGTGAGGTAACG--AAAACTTCAGTTAATAGCTGGA
Panacagrimonas perspica G soil142^{T} (AB257720)	CTTTAGGTGGGGGATAGTCCGGGGAAACTCGGT--TAGTTGGTAGGGTAATG--AAAGCTTCACCTAATACGTGGA
Algiphilus aromaticivorans DG1253^{T} (DQ486493)	CCTTGAGTGGGGGATAGCCCGGGGAAACCCGGA--TTGTTGGTGAGGTAAAG--CAAGCTCAAACTAATACTTTGA
**Out group**	
Escherichia coli JCM1649^{T} (LC069032)	CTGATGGAGGGGGATAACTACTGGAAACGGTAG--TAGTAGGTGGGGTAACG--GGGAGTAAAGTTAATACCTTTG

The DNA bases shown in orange color are different bases from the three strains, SA29-B^{T}, YU21-B and KA5-B^{T}. The DNA sequences highlighted in light green represent the sequences for which the forward and reverse primers were designed. The DNA bases in red and blue bold letters are the sequence that distinguishes the three strains, SA29-B^{T}, YU21-B and KA5-B^{T}.

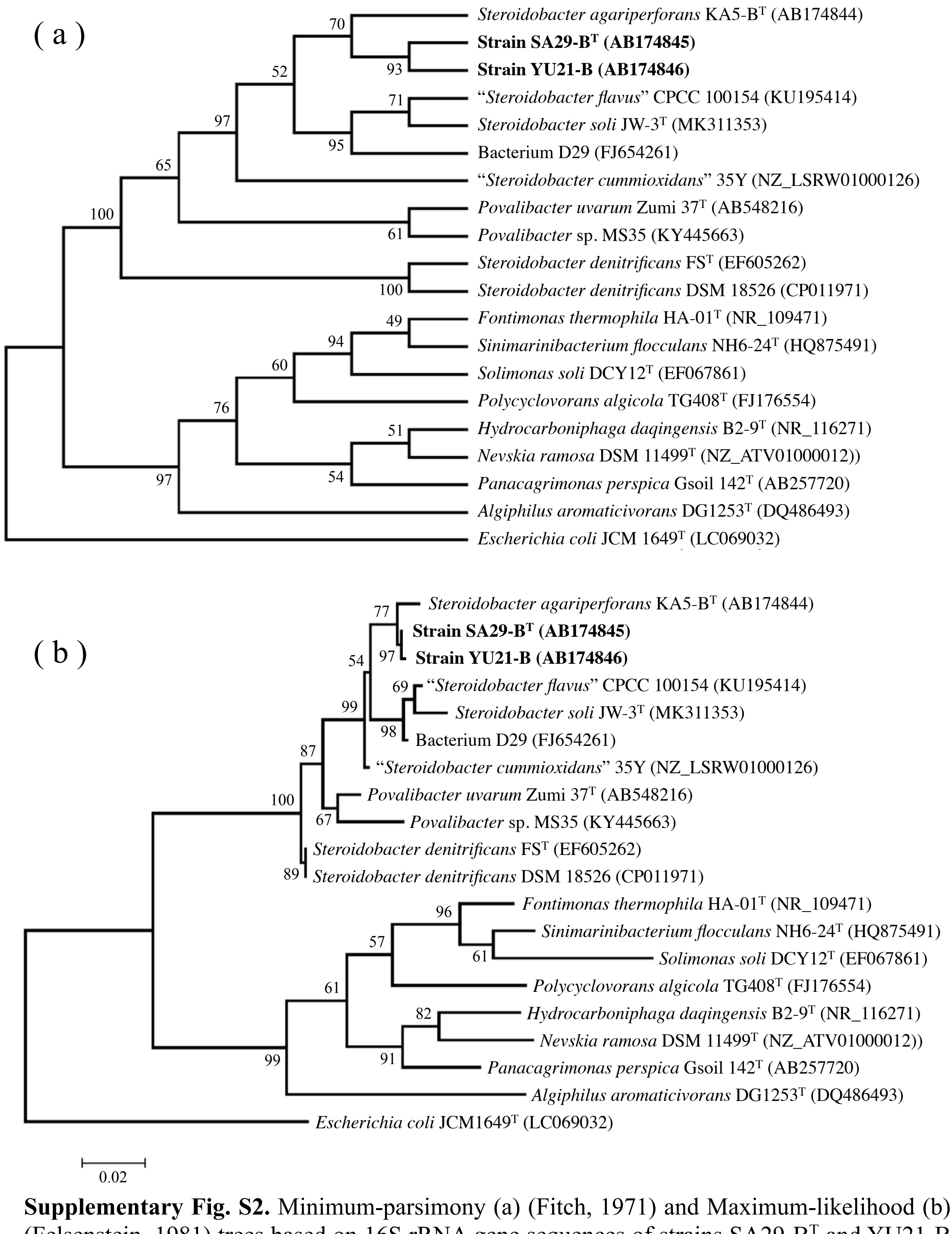

Supplementary Fig. S2. Minimum-parsimony (a) (Fitch, 1971) and Maximum-likelihood (b) (Felsenstein, 1981) trees based on 16S rRNA gene sequences of strains SA29-B^{T} and YU21-B in comparison with the closely related species within *Steroidobacteraceae* and other *Nevsliales* in *Gamma-Proteobacteria*.

Number of nodes indicates bootstrap values as calculated on the basis of 1,000 resampled dataset. Bar, 0.01 substitutions per nucleotide position. Sequences of at least 1400 nt were used for the calculation.
